# Implementing healthcare professionals’ training during COVID-19: a pre and post-test design for simulation training

**DOI:** 10.1590/1516-3180.2021.0190.R1.27052021

**Published:** 2021-08-09

**Authors:** Thiago Martins Santos, Rafaela Batista dos Santos Pedrosa, Danielle Rachel dos Santos Carvalho, Mário Henrique Franco, Juliany Lino Gomes Silva, Daniel Franci, Bruno de Jorge, Daniel Munhoz, Thiago Calderan, Tiago de Araujo Guerra Grangeia, Dario Cecilio-Fernandes

**Affiliations:** I MD, PhD. Assistant Professor, Discipline of Emergency Medicine, Department of Internal Medicine, School of Medical Sciences, Universidade Estadual de Campinas (UNICAMP), Campinas (SP), Brazil.; II RN, PhD. Assistant Professor, School of Nursing, Universidade Estadual de Campinas (UNICAMP), Campinas (SP), Brazil.; III PhD. Pharmacist and Researcher, Postdoctoral Researcher Program, Department of Medical Psychology and Psychiatry, School of Medical Sciences, Universidade Estadual de Campinas (UNICAMP), Campinas (SP), Brazil.; IV MD. Emergency Physician, Discipline of Emergency Medicine, Department of Internal Medicine, School of Medical Sciences, Universidade Estadual de Campinas (UNICAMP), Campinas (SP), Brazil.; V RN, PhD. Coordinator, Skills Laboratory, School of Medical Sciences, Universidade Estadual de Campinas (UNICAMP), Campinas (SP), Brazil.; VI MD. Hospitalist, Discipline of Emergency Medicine, Department of Internal Medicine, School of Medical Sciences, Universidade Estadual de Campinas (UNICAMP), Campinas (SP), Brazil.; VII BSc. Coordinator, Department of Academic Support, School of Medical Sciences, Universidade Estadual de Campinas (UNICAMP), Campinas (SP), Brazil.; VIII MD, PhD. Cardiologist, Discipline of Cardiology, Department of Internal Medicine, School of Medical Sciences, Universidade Estadual de Campinas (UNICAMP), Campinas (SP), Brazil.; IX MD, MSc. Trauma Surgeon, Discipline of Trauma Surgery, Department of Surgery, School of Medical Sciences, Universidade Estadual de Campinas (UNICAMP), Campinas (SP), Brazil.; X MD, MSc. Pulmonologist, Discipline of Emergency Medicine, Department of Internal Medicine, School of Medical Sciences, Universidade Estadual de Campinas (UNICAMP), Campinas (SP), Brazil.; XI PhD. Researcher, Department of Medical Psychology and Psychiatry, School of Medical Sciences, Universidade Estadual de Campinas (UNICAMP), Campinas (SP), Brazil.

**Keywords:** Coronavirus, SARS virus, Simulation training, Health Personnel, SARS-CoV-2, Simulation, Healthcare professionals, Knowledge acquisition, Airway management protocol

## Abstract

**BACKGROUND:**

Coronavirus disease-19 (COVID-19) has imposed a new reality that presents several challenges for healthcare professionals. The main challenge has been the lack of proper training in relation to an unknown disease.

**OBJECTIVE:**

To investigate healthcare professionals’ acquisition of knowledge of a new airway management protocol for COVID-19 through their participation in simulation training.

**DESIGN AND SETTING:**

Pre and post-test study with purpose sampling, carried out in a tertiary-level hospital in the city of Campinas, state of São Paulo, Brazil.

**METHODS:**

This was a cross-sectional pre and post-test intervention among healthcare professionals working in the intensive care unit and emergency department of a large hospital. The training was carried out using an ***in situ*** simulation scenario and the participants answered pre and post-tests consisting of a 20-item questionnaire about the new protocol.

**RESULTS:**

The paired-sample t test demonstrated that there was a significant increase in test score (t = −19.06; P < 0.001), from before the training (M = 8.62; standard deviation, SD = 3.53) to after the simulation training (M = 17.02; SD = 1.76).

**CONCLUSIONS:**

The simulated training had a positive impact on the healthcare professionals’ acquisition of the COVID-19 protocol. We also demonstrated that ***in situ*** simulation training was an efficient tool for implementing new protocols, thus bringing benefits to healthcare systems, professionals and patients.

## INTRODUCTION

Coronavirus disease-19 (COVID-19) has imposed a new reality through the pandemic that it has caused, and this presents challenges to healthcare professionals and systems. The high rate of transmissibility of the severe acute respiratory syndrome coronavirus 2 (SARS-CoV-2), through droplets, aerosols and contaminated surfaces, has led to development of strict protocols for individual and collective protection for patient care,^1^^,^^2^ which have been implemented throughout hospitals. Adaptations to protocols for procedures have been brought in, including use of alternative medications to minimize virus transmission in aerosol-producing procedures, donning and doffing of personal protective equipment (PPE) and other measures.

The implementation of these new protocols has required training for all front-line healthcare workers, without endangering them or their patients. Use of simulations may be an appropriate way for providing training since this replicates real-environment situations in a safe environment, and thus protects both patients and professionals from unnecessary risks. Simulation training has been widely used for continuing professional development, in order to train healthcare professionals in relation to new systems, thereby enabling them to remain up-to-date regarding new demands and protocols within their clinical practice.^3^^,^^4^^,^^5^^,^^6^^,^^7^ Simulation has played a key role in testing and implementing new workflow structures, new protocols and cognitive resources,^8^ through offering participants the possibility to practice rare and critical events in a controlled environment.^9^

In the current pandemic, simulation has been shown to be useful for testing healthcare systems, processes and new protocols.^10^^,^^11^^,^^12^^,^^13^^,^^14^^,^^15^ Moreover, studies have shown that simulation is an appropriate teaching tool that has the capacity to quickly prepare frontline teams for changes that are necessary, through generating gains in knowledge and skills.^10^^,^^13^^,^^16^^,^^17^

Healthcare professionals have become protagonists in this new reality. For safe and effective care to be developed, it necessary to train these professionals in large numbers, in settings ranging from primary care to quaternary-level hospitals. Given the high rate of transmissibility of COVID-19, simulation training can ensure individual, team and system readiness^17^ while increasing patient and professional safety.

## OBJECTIVE

To investigate healthcare professionals’ acquisition of knowledge of a new airway management protocol for COVID-19 through their participation in simulation training.

## METHODS

This was a pre and post-test study with purpose sampling that was conducted between March 31 and April 14, 2020. The data for this study came from an institutional training protocol for healthcare professionals working in the intensive care unit and emergency department of a large hospital. The simulated training was carried out *in situ* using PPE and a high-fidelity simulator. Participants answered a pre-test and a post-test consisting of 20 questions with very short answers, on donning and doffing (five questions), oxygen therapy for patients with acute respiratory distress syndrome (ARDS) due to COVID-19 (four questions), orotracheal intubation (five questions), and choice of medication for starting mechanical ventilation (six questions) (**Appendix 1**). The maximum possible score for the test was 20.

The training offered to the participants consisted of the following steps:

1. Pre-test.
2. Simulation training on donning of personal protective equipment (PPE). The instructors carried out the demonstration and gave instructions to the participants. Then, the participants performed individual practical training.
3. Participation in an interdisciplinary simulation scenario. The participants received a briefing with the necessary instructions. The objective was to teach the new COVID-19 protocol, focusing on making a clinical diagnosis of likely COVID-19 infection and recognizing acute respiratory failure, followed by use of orotracheal intubation and choosing the medication for starting mechanical ventilation. This scenario is shown in **Appendix 2**.
4. Practical training on doffing of PPE. After the scenario had been described, the participants received instructions and a demonstration of the technique, and performed individual practical training.
5. Debriefing. After the doffing training, the participants discussed and reflected on contamination, the decision to intubate and the COVID-19 protocol, guided by instructors experienced in the COVID-19 protocol and the simulation methodology.
6. Post-test.

For data analysis, we used a paired-sample t test to investigate the difference between the pre and post-test scores. The data were analyzed using IBM-SPSS version 27.0 (IBM Corporation, Armonk, New York, United States)

This study was approved by our university’s Ethics Committee (protocol number 3.943.505; date of approval March 30, 2020). Written informed consent was obtained from all participants.

The sample represented a small proportion of the total number of participants in the institutional training protocol. Due to the urgency of preparing the frontline team to face COVID-19, the managers of the hospital and the authors of this study came together to develop a training program for all doctors, nurses and physiotherapists. Since the physiotherapists were in the first training sessions, they were not included in the present study.

The training protocol started as a proposal from this hospital for coping with the COVID-19 pandemic. At a time when most of the employees had already undergone training, the authors submitted this study to the local Ethics Committee and obtained ethical approval for it during the final training phase, when a small number of employees were still waiting for training.

## RESULTS

Forty-eight professionals participated in this study. Most of them were doctors (68.7%), followed by nurses (18.8%) and nursing technicians (12.5%). Among all the participants, 79.2% were female. The participants’ ages ranged from 23 to 48 years, with an average of 31.96 years and a standard deviation of 7.11.

The paired-sample t test demonstrated that there was a significant increase in test score (t = −19.06; P < 0.001) from before the training (M = 8.62, standard deviation, SD = 3.53) to after the simulation training (M = 17.02; SD = 1.76). Significant increases from before to after the simulation training were also found in the subdomains (**Table 1**).

**Table 1:** Mean, standard deviation (SD), t and P-value of the subdomains of the knowledge test

Subjects	Donning and doffing	Oxygen therapy	Medication	Orotracheal intubation
Before Mean (SD)	2.29 (1.01)	1.81 (1.00)	2.65 (2.19)	1.87 (1.38)
After Mean (SD)	4.67 (0.59)	3.10 (0.69)	5.62 (0.84)	3.62 (0.87)
t	−15.76*	−7.23*	−10.03*	−9.12*

*P < 0.001.

## DISCUSSION

In this study, we investigated healthcare professionals’ acquisition of a new airway management protocol for COVID-19 in a large hospital. We found evidence of a significant and considerable improvement from before to after training, which was in line with previous data in the literature.^4^^–^^7^ Our data also corroborated studies that showed that simulation was essential for developing, testing, refining and implementing new workflows and protocols in healthcare.^8^^,^^10^^,^^11^^,^^18^^,^^19^ This was also essential in the context of the COVID-19 pandemic, in which we trained healthcare professionals on the new protocol for safe and effective care of patients with COVID-19.

We chose *in situ* simulation because of the necessity to train healthcare professionals while they continued to do their clinical work. Thus, we organized training sessions in the mornings, afternoons and nights. *In situ* training also decreased the circulation of healthcare professionals throughout the university, thus avoiding exposing vulnerable people to the risks of COVID-19. Furthermore, these healthcare professionals remained close to their units, which allowed them to respond to any emergency when necessary. *In situ* simulation is a fast and efficient way for training a multidisciplinary team because training takes place during the team’s hours of service, using the workplace resources.^19^

In this study, we decided to use questions with very short answers. Use of questions of this nature made it possible to assess the participants’ knowledge without giving any clues about the correct answer. This was especially important because our sample was composed of experienced healthcare professionals who would have the capacity to deduce the correct answer by looking at the alternatives. Moreover, it was easier to grade the results than it would have been if essay questions had been used. Lastly, questions with very short answers have been shown to have the same psychometric properties as standard multiple-choice questions, while avoiding recognition of the correct answer.^20^

This study had some limitations. First, the purposing sample may have limited the generalizability of our findings. Another limitation was that we used a one-group pre and post-test design. Both the sampling and the study design were selected because of the importance of training frontline workers and we designed the simulation training based on the best evidence available.

Most importantly, we demonstrated the possibility and usefulness of simulation training during COVID-19. Lastly, we focused mostly on knowledge acquisition, since all the frontline workers were skillful with regard to airway management but lacked expertise relating to the new protocol.

## CONCLUSIONS

The simulated training had a positive impact on the healthcare professionals’ acquisition of the COVID-19 protocol. We also demonstrated that *in situ* simulation training was an efficient tool for implementing new protocols, thus bringing benefits to healthcare systems, professionals and patients.

## Appendix 1

**Table:** Questions: pre and post-test

**Donning and doffing** 1 - During the donning, when should I put on the respiratory protection mask (PFF2/N95)? 2 - During the donning, when should I put on the gloves? 3 - How many times should hand hygiene be done BEFORE the donning? 4 - How many times should hand hygiene be done DURING the doffing? 5 - What are the necessary PPE for the intubation of a suspected COVID-19 patient?
**Oxygen therapy for patients with severe acute respiratory syndrome (SARS) due to COVID-19:** 6 - The device to be initially used to provide oxygen therapy to patients with SARS due to COVID-19 is: 7 - What is the oxygen saturation target to be obtained through initial oxygen therapy, for patients with COVID-19 and respiratory failure? 8 - Which device should be used for pre-oxygenation of a patient with suspected COVID-19? 9 - Which device should be installed between the mask and the bag, before pre-oxygenation, to minimize aerosol dispersion?
**Choice of medication for rapid sequence intubation (RSI):** 10 - What is the recommended drug for RSI in a patient with suspected COVID-19? 11 - How long should pre-oxygenation be performed on a patient with suspected COVID-19? 12 - Which drug is indicated for premedication before intubation? 13 - Which drug is indicated for induction (hypnosis)? 14 - Which drug is indicated for neuromuscular block? 15 - What is the average time needed for the premedication drug to be effective before administration of hypnosis?
**Orotracheal intubation** 16 - Since ventilation with bag-valve-mask (BVM) should be avoided among COVID-19 patients, how should they be ventilated when checking the orotracheal tube position? 17 - Since ventilation with BVM should be avoided among COVID-19 patients, which alternative method for checking the tube position can be used, if available? 18 - What is the potential complication of the tube clamping technique in the case of a patient with increased airway reactivity? 19 - After intubation, mechanical ventilation must be started. Which items should be attached between the tube and the Y-piece (which connects the inspiratory and expiratory limbs of the ventilator)? 20 - Which tidal volume should be set on the mechanical ventilator?

## Appendix 2

**Table:** Scenario of severe acute respiratory syndrome (SARS) due to coronavirus disease-19 (COVID-19).

Scenario title	Severe acute respiratory syndrome (SARS) due to coronavirus disease-19 (COVID-19)
**Learning objectives** - To recognize respiratory failure in a patient with severe acute respiratory syndrome (SARS); - To begin treatment for respiratory failure; - To recognize failure of the initial treatment; - To intubate the patient in the event of oxygen therapy failure.	
**Materials** - One high-fidelity mannequin; - One multiparametric monitor; - One gas panel; - Oxygen therapy devices - One airway material kit - Infusion therapy devices; - Water ampoules for injection, with the following identifications (one each): fentanyl, etomidate, midazolam, succinylcholine, ketamine, rocuronium, salbutamol, magnesium sulfate, dexamethasone, hydrocortisone and adrenaline	
**Scenario overview** The patient Alberto Ramos de Miranda, 52 years old, with a history of hypertension and type 2 diabetes, sought the emergency department with a complaint of four days of fever, dry cough, headache, diarrhea and dyspnea (which he noticed one day ago). The patient was first evaluated in the emergency department triage room and was referred to the emergency room (ER) due to severe hypoxemia and tachypnea. In the ER, the patient will not have an adequate (satisfactory) response to oxygen therapy, with maintenance of hypoxemia and elevated breathing effort, and with the need to institute invasive mechanical ventilation. Healthcare professionals should recognize the patient’s respiratory failure and perform orotracheal intubation due to the failure of oxygen therapy.	
**Participants**	
**Role in the scenario /participant**	**Quantity**
Physician	1
Nurse	1
Nursing technician	1
Physiotherapist	1
***Briefing*** You are a physician, nurse, nursing technician or physiotherapist in the emergency room. Upon entering the scene, you will receive the patient Alberto Ramos de Miranda, 52 years of age, who was brought in from the triage room with a main complaint of dyspnea. The patient has peripheral oxygen saturation measured at 86% and is under precaution regarding contact due to reports of flu-like symptoms preceding the current condition. You must perform patient care, handling the situation in the most appropriate way. Consider yourself donning to avoid droplets and aerosols.	
**History of the actor/mannequin** Alberto Ramos de Miranda, 52 years old, is undergoing treatment for hypertension and type 2 diabetes with hydrochlorothiazide and metformin. Four days ago, he noticed the start of a low fever, dry cough, malaise, body pain, and headache. He used analgesics, but without improvement of the condition and with worsening of symptoms. One day ago, he started to get tired with small efforts, so he came to the hospital today because he felt tired even when resting. He also reported having diarrhea and mild abdominal pain during this period. He says that he does not have any history of smoking or drinking. There are no other known comorbidities.	
**Initial programming of the mannequin** Sinus rhythm; HR 114 bpm; RR 34 bpm; SpO2 86%; BP 168 x 96 mmHg	
